# What’s love got to do with it? Relationship quality appraisals and quality of life in couples facing cardiovascular disease

**DOI:** 10.1080/21642850.2023.2237564

**Published:** 2023-07-18

**Authors:** Karen Bouchard, Alexandre Gareau, Paul S. Greenman, Kathleen Lalande, Karolina Sztajerowska, Heather Tulloch

**Affiliations:** a Prevention and Rehabilitation Center, Division of Cardiac Prevention and Rehabilitation, University of Ottawa Heart Institute, Ottawa, Canada; b Faculty of Medicine, University of Ottawa, Ottawa, Canada; c Laval University, Laval, Canada; d Université du Québec en Outaouais, Gatineau, Canada; e Monfort Hospital, Ottawa, Canada

**Keywords:** Cardiovascular disease, couples, relationship quality, quality of life

## Abstract

**Objective:**

Changes in couples’ relationship quality are common post-cardiac event but it is unclear how relationship quality is linked to patients’ and spouses’ quality of life (QoL). The purpose of the present study was to examine the association between relationship quality on QoL in patient-spouse dyads within six months of a cardiac event.

**Methods:**

Participants (N = 181 dyads; 25.9% female patients), recruited from a large cardiac hospital, completed validated questionnaires measuring demographic, relationship (Dyadic Adjustment Scale; DAS) and QoL variables (Heart-QoL & Quality of life of Cardiac Spouses Questionnaire). An Actor-Partner Interdependence Model was used to investigate actor (i.e. responses influencing their own outcome) and partner effects (responses influencing their partner’s outcome) of relationship quality and QoL.

**Results:**

Patients’ and spouses’ perceptions of relationship quality were in the satisfied range (DAS > 108; 65% of sample) and, as expected, patients reported lower general physical QoL than did their spouse (*t*_(180)_ = −10.635, *p* < .001). Patient and spouse relationship quality appraisals were positively associated with their own physical (patient *β *= .25; spouse *β *= .05) and emotional/social (patient *β *= .21; spouse *β *= .04) QoL. No partner effects were identified.

**Conclusion:**

High quality relationship appraisals appear to matter for patients’ and spouses’ QoL after the onset of CVD.

## Acronyms listed in text

CVD = cardiovascular disease; QoL = quality of life; APIM = Actor Partner Interdependence Model; DAS = Dyadic Adjustment Scale; Heart-QoL = Heart-Related Quality of Life; QL-S*P* = Quality of Life of Cardiac Spouses; SEM = Structural Equation Model; MCAR = missing completely at random.

## Introduction

Cardiovascular disease (CVD) is a life-limiting condition and is one of the most common chronic diseases in the United States, accounting for one in every four deaths. The clinical course of CVD can be unpredictable and associated with the burden of physical and emotional symptoms, loss of independence, and disruptions to social roles, all of which have the potential to severely degrade patients’ quality of life (QoL). Health-related QoL is a key patient-reported health endpoint that quantifies the limitations that a condition imposes on the ability to function as desired. (Ski & Thompson, 2010; Spertus, 2008) Although QoL is recognized as a significant health outcome on its own, several lines of research have demonstrated that QoL is a robust predictor of reduced readmissions (Vámosi et al., 2020) and recurrent major adverse cardiovascular events, (Mommersteeg et al., 2009) and is correlated to the uptake of preventative health behaviors that ameliorate cardiovascular risk and improve recovery. (Kang et al., 2017) Therefore, identifying influencing factors related to QoL may provide effective strategies for disease management (Sajobi et al., 2018) and reduced health care expenditure.

Abundant research now indicates that social factors are linked to QoL post-cardiac event. For example, functional health is worse among patients with CVD who are unmarried, socially isolated, lonely, and those who have low perceived social support. (Golaszewski et al., 2022; Manemann et al., 2018; Valtorta et al., 2016) These social factors represent the structural (e.g. marital status) and functional (e.g. perceived social support) aspects of social relationships, but accumulating research from relationship science indicates that it is not just the presence, but the quality of these relationships that are consequential for optimal recovery. (Valtorta et al., 2016) Indeed, the effect sizes of marital quality are roughly equivalent to those of diet and exercise on cardiovascular disease outcomes and mortality. (Robles et al., 2014) The connection between relationship quality (i.e. feelings of satisfaction with the relationship, positive attitudes towards one’s partner, and minimal hostility in couple interactions), a subjective qualitative evaluation, and QoL specifically, remains poorly understood. This is an important avenue for research as reductions in relationship quality are pervasive post-cardiac-event. (Hsieh & Hawkley, 2018) To date, only four studies have been conducted connecting relationship quality to QoL in cardiac populations. (Molloy et al., 2008; Rohrbaugh et al., 2008; Roijers et al., 2016; Yu & Zhang, 2020) These studies observed that relationship quality is associated with QoL cross-sectionally or overtime (baseline [hospitalization, early discharge] to 6 months-4 years). Methodological weaknesses exist, however. First, insights were derived from small sample sizes and assessments of relationship quality were ascertained from just one member of the couple. Investigating perceptions from both participants is more in line with an interactionist perspective (i.e. individuals influence their own and others’ outcomes) (Kelley & Thibaut, 1978) and several decades of research stemming from relationship science that notes the causal influences of spousal outcomes. (Bidwell et al., 2017; Bouchard et al., 2019; Dalteg et al., 2011; Kiecolt-Glaser & Wilson, 2017; Randall et al., 2009; Wittenberg & Prosser, 2016) If relationship quality assessments from both members of the dyad are linked to QoL, targeting relationship quality with patients and spouses within secondary prevention programming may be a promising avenue for optimizing couples’ health in the aftermath of a CVD diagnosis. To inform intervention need and potential targeted outcomes, an important starting point is to examine associations among relationship quality and QoL among larger samples of patients with CVD and their spouses.

In this study, relationship quality and QoL were examined in patient-spouse dyads, in which one partner experienced a cardiac event in the last 6 months, using an Actor-Partner Interdependence Model (APIM). (Cook & Kenny, 2005) In this method, patient and partner variables are taken into account using a single Structural Equation Model (SEM) to reveal actor (i.e. responses influencing their own outcome) and partner (i.e. responses influencing the other’s outcome) effects. (Gareau et al., 2016) Specifically, it was hypothesized that patients’ and spouses’ perceptions of relationship quality would be associated with their own QoL (actor effects) and the other’s QoL (partner effects).

## Methods

Participants were recruited from the cardiac rehabilitation program at a large tertiary cardiac hospital in Canada. To be eligible, participants had to: (a) have directly (i.e. patient) or indirectly (i.e. spouse) experienced a ‘cardiac event’ in the previous six months such as heart failure, myocardial infarction, angina, or hospitalization for cardiovascular-related procedure (e.g. percutaneous coronary intervention, coronary artery bypass grafting); (b) be in a couple relationship (i.e. married, common law, in a committed relationship) for at least one year; (c) be living in the same household for at least one year; (d) be 18 years of age or older; (e) be able to read and/or speak in English or French; and, (f) be willing to provide informed consent. This study was approved by the Ottawa Health Science Research Network Ethics Board. Sample size calculations were based on the primary objective, relationship quality, using the Dyadic Adjustment Scale (DAS), and to ensure sufficient statistical power for the APIM. Eligibility was ascertained by the screening of medical records and confirmed with the patient at an appointment or by phone. Research staff then met with the patient at their onsite appointment to obtain written informed consent. If spouses were not present, patients brought home the consent form and questionnaires. Participants completed the measures, independently, onsite or at home, and returned the questionnaires to the study site.

### Measures

**Demographic questionnaire.** All participants completed a medical and sociodemographic questionnaire gathering personal, relationship, and clinical information. Patients’ cardiac diagnoses and medical history were confirmed via patients’ medical charts. **Relationship quality.** The DAS (Spanier, 1976) is a validated 32-item, self-report questionnaire that measures couple satisfaction, cohesion, consensus, and affectionate expression. Total Scores ≥108 indicate couple satisfaction; scores ≤107 indicate distress. The psychometric properties of the DAS are well established (Spanier, 1976; Spanier & Thompson, 1982), including in populations with chronic disease. In this study the internal consistency was acceptable (patient; α = .927, partner; α = .920). **Quality of life.** Patients completed the Heart-Related Quality of Life Scale (Heart-QoL) (Oldridge et al., 2014b; Oldridge et al., 2014a; Oldridge et al., 2018) a 14-item disease-specific QoL scale (α = .871). Questions are used to assess feelings on how cardiac disease affects daily functioning over the last four weeks. It provides a global health related QoL score as well as physical and emotional subscales. This scale is a reliable and valid measure among cardiac populations in over 30 languages. (Oldridge et al., 2014b; Oldridge et al., 2014a) Partners completed the Quality of Life of Cardiac Spouses Questionnaire (QL-SP), (Ebbesen et al., 1990) a 26-item measure used to assess emotional functioning and combined physical and social functioning (α = .907). It has strong psychometric properties. (Thomson et al., 2013) Higher scores on the Heart-QoL and QL-SP indicate better QoL.

### Data analysis

An ‘SEM-APIM’ (Cook & Kenny, 2005) was used to uncover unique actor and partner cross-sectional effects of relationship quality and QoL. Missing data points were handled through a multiple imputation procedure using the iterative Markov chain Monte Carlo method in SPSS (version 26). Ten datasets were imputed at the item-level and then for each imputed dataset scale scores were calculated. Main analyzes were all performed with Mplus 8.4, which provide pooled estimates. A Little’s test revealed that the pattern of missing data (*M* = 2.3%, *SD* = 2.5%, min = 0%, max = 16%) was not different from a completely random pattern (*MCAR χ^2^*_(8845)_ = 8961.839, *p* = .190).

## Results

**Figure 1.**
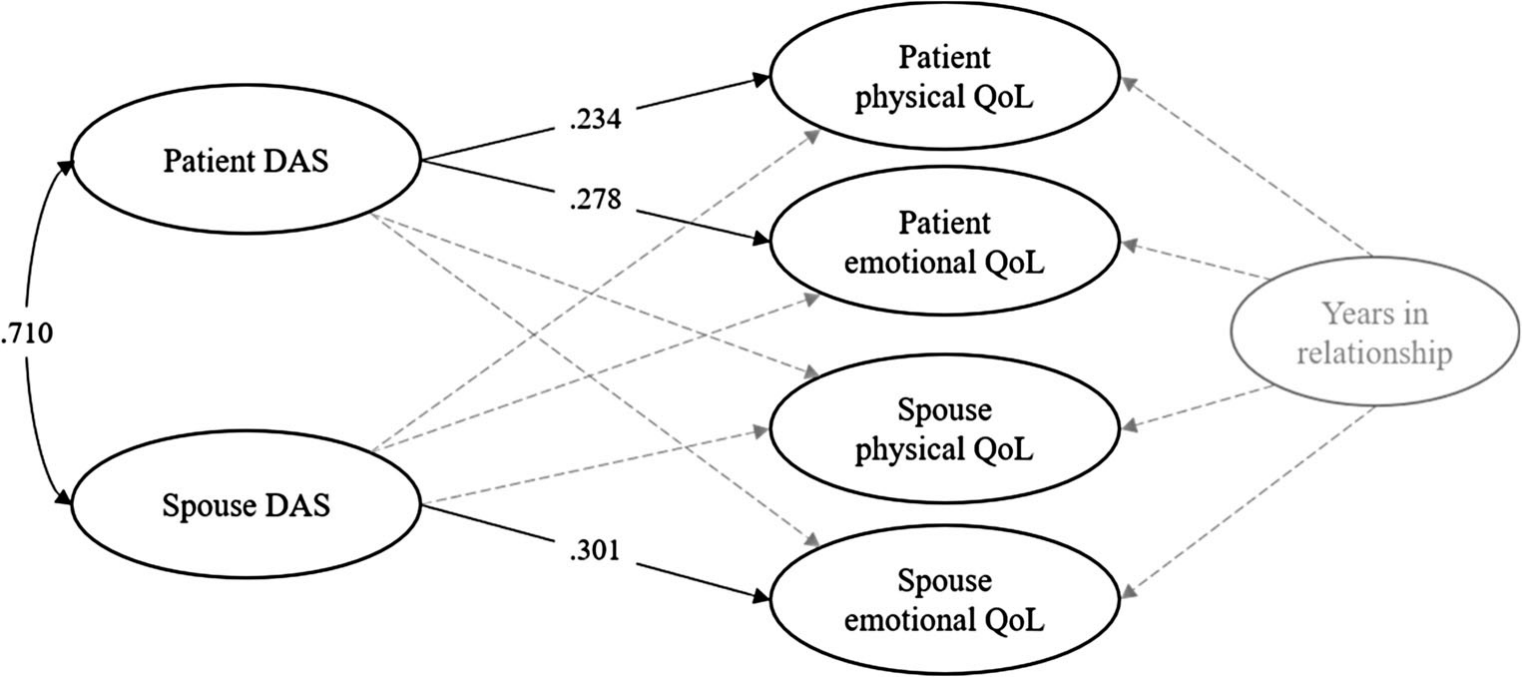
The standard saturated cross-sectional SEM-APIM. Significant standardized regression coefficients are presented with bold lines. Non-significant regression coefficients are omitted but presented with grey dash lines. Years in relationship is also freely correlated with patient and spouse relationship quality, but not presented in this visualization. Outcome residual variances are all freely correlated.

Patients and spouses who met the eligibility criteria were approached to participate (*N *= 309); 83% (*N *= 257 dyads) consented to participate and 58.6% (*N *= 181 dyads) returned completed questionnaires (70.4% of couples who gave their consent). Please see the participant flow chat in the online supplemental material. Patients were predominantly male (74.1%) and in heterosexual relationships. Patients’ average age was 64.6 years (*SD* = 9.99); partners were close in age (*M* = 61.6 years, *SD* = 10.49) (Table 1). The descriptive statistics and bivariate associations for relationship quality and QoL are provided in Table 2. Most patients’ and spouses’ perceptions of relationship quality were in the satisfied range (DAS >108; 65% of sample). Patient and spouse relationship quality scores were not statistically different (*diff* = 1.415, *se* = .982, *p* = .150) and were highly correlated (r = .71), indicating a high level of within-dyad similarity. As expected, patients reported lower general physical QoL than did their spouses (*t*_(180)_ = −10.635, *p* < .001). The standard SEM-APIM revealed three actor effects but no partner effects (Figure 1). The standardized regression coefficients from the saturated SEM-APIM are presented in Table 3; years in relationship was controlled for in the model. Patient relationship quality was positively associated with patient physical and emotional QoL. Spouse relationship quality was positively associated with spouse emotional QoL.

**Table 1.** Participant Demographic and Clinical Information.

Characteristic	Total (N = 181)
**Age Patient** (M, SD)	64.6 (9.99)
**Age Partner** (M, SD)	61.76 (10.49)
**Sex Patient** (n, % female)	47 (25.9)
**Sex Partner** (n, % female)	134 (74.1)
**Relationship Status** (n, %)	
Married	153 (85.0)
Common Law	27 (15.0)
**Years in Relationship** (M, SD)	33.93 (14.87)
**Ethnicity** (n, %)	
White	163 (91.1)
Black	3 (1.7)
Latin/Hispanic	2 (1.1)
Asian	7 (3.9)
Middle Eastern	1 (0.6)
Aboriginal	1 (0.6)
Other	2 (1.1)
**Education** (n, %)	
Elementary school	1 (0.6)
High School	49 (27.4)
College Degree	47 (26.3)
University Degree	81 (45.3)
**Diagnosis of Patient** (n, %)	
Coronary Artery Disease	120 (66.3)
Arrhythmia	15 (8.3)
Congestive Heart Failure	21 (11.6)
Valve Disease	20 (11.0)
Congenital Heart Disease	2 (1.1)
Aortic Aneurysm	3 (1.7)

Note*.* %, percentage; n, number; M, mean; SD, standard deviation.

**Table 2.** Descriptive and bivariate associations.

Variables	M	SD	1	2	3	4	5	6
**1. Patient DAS**	114.783	17.461						
**2. Spouse DAS**	113.368	16.980	.710*					
**3. Patient physical QoL**	1.838	.678	.102	-.019				
**4. Patient emotional QoL**	2.161	.715	.248*	.144	.327*			
**5. Spouse physical QoL**	5.186	.931	.064	.093	.339*	.147*		
**6. Spouse emotional QoL**	4.939	.924	.094	.211*	.130	.078	.646*	
**7. Years in relationship**	33.649	14.526	.122	.024	.022	.102	.085	.077

Note. * *p *< .05, DAS = dyadic adjustment scale, QoL = Quality of life

**Table 3.** Standardized regression coefficients from the saturated SEM-APIM.

	Patient outcomes				Spouse outcomes			
	Physical QoL		Emotional QoL		Physical QoL		Emotional QoL	
Effect	*β*	*se*	*β*	*se*	*β*	*se*	*β*	*se*
Patient DAS	.234*	.101	.278	.122	-.022	.104	-.130	.098
Spouse DAS	-.184	.107	-.055	.111	.106	.111	.301*	.121
Years in relationship	-.002	.077	.069	.071	.085	.071	.085	.073

Note*.* * *p* < .05, DAS = dyadic adjustment scale, QoL = Quality of life

## Discussion

This research indicated that patients’ relationship quality appraisals were significantly related to their own physical and emotional QoL. Similarly, spouses’ perceptions of relationship quality were significantly related to their own emotional QoL. These actor effects indicate that high quality relationships seem to matter for patients’ and spouses’ QoL after the onset of CVD. The identified actor effects are consistent with our hypotheses and with an integrative review (Hooker et al., 2015) that determined that better relationship quality was related to reduced patient mortality, increased health status, and less distress in both patients and caregivers. No studies profiled within the review, however, used APIM to analyze dyadic effects. One study examined relationship quality and QoL among both partners in the relationship, (Rohrbaugh et al., 2008) but had a small sample size (*N *= 57 dyads) and examined effects among patients with heart failure, a severely life limiting disease, precluding generalizations to other CVD diagnoses that may be punctuated less by periods of acute disease progression than seen in heart failure. An additional three studies examined the effects of marital relationship quality and QoL, but only among patients with CVD, not spouses. (Molloy et al., 2008; Roijers et al., 2016; Yu & Zhang, 2020) Specifically, Roijers and colleagues (Roijers et al., 2016) observed that patients with less optimal relationship quality did not reach the QoL norms (mental component summary scores) of a healthy population. In the remaining studies, marital relationship quality predicted reduced mental QoL at 12-months post-diagnosis (Molloy et al., 2008) and increases in patients’ functional limitations at the 2-year and 4-year follow up (Yu & Zhang, 2020) were observed. Results from the current study are from a larger sample in which perceptions of relationship quality and health from both members of the dyad were considered, albeit cross-sectionally. Considering the previous literature in combination with the current study results, it appears that relationship quality may be an important associative and predictor variable in the QoL of both patients and spouses post-cardiac event.

There were no statistically significant partner effects identified in this study when using the APIM. This indicates that a partner’s appraisal of the relationship may not be linked to the other partner’s QoL. This result was surprising, given that previous literature examining psychosocial outcomes among cardiac couples found statistically significant partner effects using APIM. (Bouchard et al., 2021; Bouchard et al., 2021; Chung et al., 2009; Dalteg et al., 2016; Thomson et al., 2020) The absence of partner effects in this study may be partially explained by the high levels of correlation between patients’ and spouses’ DAS scores. It is also possible that relationship appraisals are more influential to patients’ and spouses’ QoL when these assessments are similarly high. From a clinical perspective, alignment in patients’ and spouses’ perceptions of important facets of their relationship might reduce emotional disconnection or conflict and enhance openness, responsiveness, and effective communication, leading to better overall physical, emotional, and social health. (Gottman & Gottman, 2017; Novak et al., 2014) Simply stated, if couples are on the same page about their relationship, they may be more likely to come together to manage CVD, and therefore to have better QoL outcomes.

This study is limited by a sample that was largely homogeneous (e.g. heterosexual, white, well-educated, and male patients) and recruited from one geographical area. Furthermore, most couples in this sample reported high relationship satisfaction, leading to a potential ceiling effect. It is possible that negative relationship quality is more significantly associated with functional limitations than positive relationship quality is with optimal functional health. (Yu & Zhang, 2020) It is also possible that other psychosocial constructs seminal to understanding relationship functioning, such as attachment orientations (George-Levi et al., 2020; Pietromonaco et al., 2013) or dyadic coping, (Kayser et al., 2018) may be more potent predictors of QoL among patients and spouses confronting chronic disease. Future research could consider utilizing the DAS as well as other measures of relationship functioning among couples with more relationship distress and lower levels of QoL. The two QoL measures used in this study are comprised of different subscales (i.e. physical and emotional QoL for the Heart QoL [patients] and emotional, physical/social for the QL-SP [spouses]). Future studies could supplement the disease specific QoL measures with a general QoL measure (e.g. SF-36) used for both patients and spouses. (McHorney et al., 1993) Most importantly, inferences of temporality or causality cannot be concluded in this cross-sectional research. Despite these limitations, the findings indicate that there is merit in pursuing additional resource-intensive research (prospective longitudinal or controlled studies) examining the role of relationship quality and QoL among cardiac couples. Targeting relationship quality in secondary prevention programs (e.g. learning to identify relationship patterns, increasing responsiveness) may be a fruitful approach for improving patients’ and spouses’ QoL, but more evidence is needed to inform such programming.

## Author contribution

KB, PG, KS, and HT contributed to the conception and design of the project. KB, AG, KL, KS, and HT contributed to the acquisition, analysis and interpretation of the data for the work. KB and AG drafted the manuscript and PG, KL, KS, and HT critically revised the manuscript. All gave final approval and agree to be accountable for all aspects of work ensuring integrity and accuracy.

## Compliance with ethical standards

This study was approved by the Ottawa Health Science Network Research Ethics Board (Protocol #: 20180793-01H). Written informed consent was obtained from all participants before participating in the study. Participants signed informed consent regarding publishing their data.

## Data Availability

Data informing the article will be made accessible upon manuscript acceptance.
